# Highly Thermally Conductive Adhesion Elastomer Enhanced by Vertically Aligned Folded Graphene

**DOI:** 10.1002/advs.202201331

**Published:** 2022-10-17

**Authors:** Huitao Yu, Yiyu Feng, Can Chen, Heng Zhang, Lianqiang Peng, Mengmeng Qin, Wei Feng

**Affiliations:** ^1^ Tianjin Key Laboratory of Composite and Functional Materials School of Materials Science and Engineering Tianjin University Tianjin 300350 P. R. China; ^2^ Key Laboratory of Materials Processing and Mold Ministry of Education Zhengzhou University Zhengzhou 450002 P. R. China

**Keywords:** adhesive, elastic, self‐learning and distinguishing, thermally conductive

## Abstract

Heat and stress transfer at an interface are crucial for the contact‐based tactile sensing to measure the temperature, morphology, and modulus. However, fabricating a smart sensing material that combines high thermal conductivity, elasticity, and good adhesion is challenging. In this study, a composite is fabricated using a directional template of vertically aligned folded graphene (VAFG) and a copolymer matrix of poly‐2‐[[(butylamino)carbonyl]oxy]ethyl ester and polydimethylsiloxane, vinyl‐end‐terminated polydimethylsiloxane (poly(PBA*x*‐*ran*‐PDMS)). With optimized chemical cross‐linking and supermolecular interactions, the poly(PBA‐*ran*‐PDMS)/VAFG exhibits high thermal conductivity (15.49 W m^−1^ K^−1^), an high elastic deformation, and an interfacial adhesion of up to 6500 N m^−1^. Poly(PBA‐*ran*‐PDMS)/VAFG is highly sensitive to temperature and pressure and demonstrates a self‐learning capacity for manipulator applications. The smart manipulator can distinguish and selectively capture unknown materials in the dark. Thermally conductive, elastic, and adhesive poly(PBA‐*ran*‐PDMS)/VAFG can be developed into core materials in intelligent soft robots.

## Graphical abstract

With optimized chemical cross‐linking and supermolecular interactions, the poly(PBA‐*ran*‐PDMS)/VAFG composite exhibits high thermal conductivity, elastic, and interfacial adhesion. Manipulator equipped with poly(PBA‐*ran*‐PDMS)/VAFG is highly sensitive to temperature and pressure, and demonstrates a self‐learning, distinguishing unknown materials in the dark.

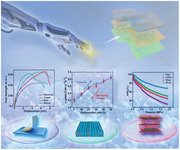

## 1 Introduction

Bionic tactile perception is a core technology for intelligent robots exploring extreme or less‐accessible territories. Among several devices, the non‐visual flexible tactile sensor has demonstrated considerable potential for discovering and identifying unknown objects owing to its ability to sense a range of physical characteristics, including size, shape, temperature, and hardness.^[^ ^1^ ^]^ Tactile sensor‐led information harvesting further enables multifunctional and intelligent robots to execute the tasks of capturing, shifting, and avoiding objects. In general, temperature, morphology, and modulus (hardness) are physical characteristics used to analyze and identify objects.^[^ ^2^ ^]^ Such characteristics depend on heat and stress transfer at the interface between the target object and the sensing material based on intimate contact.

Interactions between materials in contact are critical for perceptual sensitivity to heat and stress at the interface. However, the difference in chemical structure and surface morphology between the target object and the sensing material generally leads to weak interactions, which decreases the effective contact area. Consequently, stress transfer at the interface is limited, particularly for a rough or irregularly shaped surface, and interfacial separation lowers heat conduction. To overcome these limitations, a previous study focused on fabricating a temperature sensor using highly elastic polymers that increase the effective contact area and favor interactions under applied pressure.^[^ ^3^ ^]^ However, high‐resolution sensing is restricted after thousands of cycles due to permanent plastic deformation during stretching/shrinking. Moreover, an elastic polymer generally displays a relatively low thermal conductivity (***k***) and large heat loss in contact (area or strength), which results in a temperature difference between the target material and the sensor. The deviation in temperature decreases both sensitivity and accuracy. Furthermore, adhesion between the object and the target material is another important property for robots as it affects temperature sensing, capturing, shifting, or avoiding the target object.^[^ ^4^ ^]^ However, current elastic polymers used in flexible sensor exhibit low adhesive strengths toward several materials, and interfacial contact stress changes during compression resilience. These results indicate that interfacial materials combining elasticity, adhesion, and heat conduction are beneficial for increasing sensing performance and enabling object‐capture behavior.

Thermally conductive, elastic, and viscous polymer‐based materials are ideal interfacial‐material candidates for multifunctional flexible tactile devices.^[^ ^5^ ^]^ However, combining efficient phonon transport, good elasticity, and high adhesion force is a challenge owing to the trade‐off between structural cross‐linking and chain orientation. Specifically, a high adhesion force arises from the formation of strong physical and chemical bonds via molecular interaction (such as hydrogen bonds and electrostatic and van der Waals forces), and chain stretching controlled by weak cross‐linking causes elasticity.^[^ ^6^ ^]^ Therefore, optimizing supermolecular interactions and suitable cross‐linked chains is an effective method for endowing polymers with elasticity and adhesiveness. However, the random cross‐linked structure in three dimensions prevents harmonic lattice vibrations, and consequently, phonon scattering. Consequently, a relatively low thermal conductivity ***k*** value impedes heat conduction through the random polymer chain.

Introducing 3D, thermally conductive fillers into polymers is an effective strategy for increasing the ***k*** value. The rigid crystalline structure of an individual filler favors phonon transport but results in a decrease in both elasticity and adhesion of the composites owing to low stretching and poor chemical activity.^[^ ^7^ ^]^ Therefore, only small concentrations of fillers can be incorporated into elastic and viscous polymers, and the composites generally show low ***k*** values owing to the high interfacial heat resistance between fillers and polymers. The fracture toughness and thermal conductivity of the material can be remarkably increased at the expense of elasticity and adhesion force. Zhang et al.^[^ ^8^ ^]^ synthesized vertically aligned graphene tube/polydimethylsiloxane composites using chemical vapor deposition, with a ***k*** value of 1.7 W m^−1^ K^−1^ reported at 4.5 wt% graphene. Shen et al.^[^ ^9^ ^]^ developed a highly resilient, strongly and interfacially bonded, thermally conductive composite by bonding silica‐coated graphene nanoparticles (silica@GNPs) with a polydimethylsiloxane matrix. The composite exhibited a ***k*** value of 0.497 W m^−1^ K^−1^ at a filler content of 2 wt%. Jeong et al.^[^ ^10^ ^]^ reported mechanically stretchable and electrically insulating elastomer composites with embedded liquid metal inclusions. The in‐plane thermal conductivity of the composite (75 wt%) was 0.6 W m^−1^ K^−1^, and it also can enhance thermal response or efficiency of soft and stretchable thermal devices or systems. Bartlett et al.^[^ ^11^ ^]^ achieved an increase in thermal conductivity (4.7 ± 0.2 W·m^−1^·K^−1^) over the base polymer under stress‐free conditions by incorporating liquid metal microdroplets into a soft elastomer. High resilience can effectively improve the interfacial compatibility and heat transfer efficiency of a composite. However, the ***k*** values of reported composites are relatively low. Due to low composite surface adhesiveness and poor macro‐contact between materials, thermally conductive materials are rarely used in flexible contact‐based tactile sensing with recognition functions.

In this study, we synthesized a series of polymeric materials with high adhesion and high elongation by optimizing high‐density hydrogen bonding (H‐bonding) interactions and strong cross‐linking between molecules. polydimethylsiloxane, vinyl‐end‐terminated (PDMS) was used as the cross‐linking reinforcer, and poly‐2‐[[(butylamino)carbonyl]oxy]ethyl ester (PBA) was used as the soft segment. A new tactile perception composite (poly(PBA‐*ran*‐PDMS)) with strong interfacial adhesion, high elasticity, and a high ***k*** value was prepared by a physical impregnation process under vacuum conditions based on a force–thermal coupling design concept using poly(PBA‐*ran*‐PDMS) as the polymer matrix and VAFG as the thermally conductive filler. Poly(PBA‐*ran*‐PDMS)/VAFG can provide a theoretical basis and technical support for the design and preparation of future high‐performance polymer‐based tactile perception composites. In the application of these composite materials, the core technologies that we examined are temperature sensing. Section 2 of the paper is divided into the following sections: Preparing and optimizing the properties of the polymers, morphologies and mechanical properties of the composites, theoretical analysis and simulating ***k*** values and the interfacial thermal resistances of the materials, manipulator self‐learning based on temperature and pressure, and identifying and sensing materials.

## 2 Results and Discussion

### 2.1 Mechanical and Adhesive Properties of Poly(PBA*x*‐*ran*‐PDMS)

**Figure 1:**
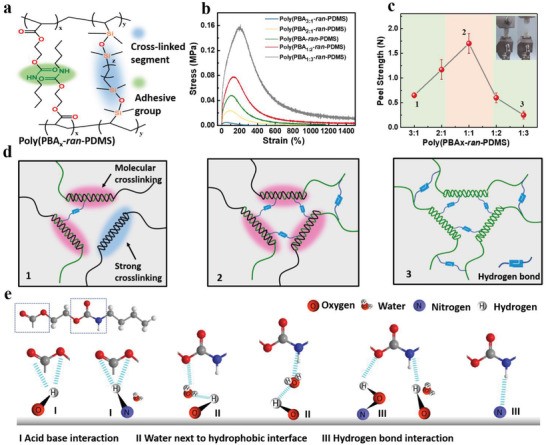
a) Molecular structures of poly(PBA*x*‐*ran*‐PDMS) block copolymers. b) Typical stress–strain curves of poly(PBA*x*‐*ran*‐PDMS) (*x* = 3:1, 2:1, 1:1, 1:2 and 1:3). The inset shows the corresponding modulus of elasticity. c) Adhesion strength of poly(PBA*x*‐*ran*‐PDMS) (*x* = 3:1, 2:1, 1:1, 1:2 and 1:3). The inset shows photographic images of the self‐adhesive tension of material fracture. d) Self‐adhesion and strength mechanism of polymers with different ratios. e) Adhesion mechanism model of poly(PBA‐*ran*‐PDMS) on the matrix.

Polymeric materials with high strengths, elongations, and adhesiveness are critical for emerging fields such as electronics, energy, advanced manufacturing, and smart robotics.^[^ ^12^ ^]^ However, the adhesion and mechanical properties of soft materials are affected by molecular cross‐linking and molecular chain motion, leading to a trade‐off between mechanical properties and adhesion. For example, PBA has excellent elongation and adhesion properties but both poor strength and plasticity. The cross‐linked network structure with special properties was obtained by incorporating different molar ratios of flexible PDMS chain units with strong cross‐linking into PBA chains with high‐density H‐bonds and adjusting the internal/external H‐bonds and electrostatic interactions (**Figure** **1**a). And the ^1^H NMR spectra of poly(PBA*x*‐*ran*‐PDMS) in CDCl_3_ was shown in Figure S1, Supporting Information. The formation of hydrogen bonds has less change in the energy, bond length, and electron cloud density. It is generally believed that the formation of hydrogen bonds is mainly due to the electron transfer from the proton acceptor to the proton donor, which leads to an increase in the bond length of the hydrogen bond and a decrease in the frequency of the stretching vibration (redshift of the hydrogen stretching vibration). Therefore, redshift is considered to be an important feature of hydrogen bonding. To verify the existence of hydrogen bonds, poly(PBA*x*‐*ran*‐PDMS) was characterized by FTIR (Figure S2, Supporting Information). The FTIR of PBA shows a characteristic peak of —N—H— at 3358 cm^−1^, and poly(PBA*x*‐*ran*‐PDMS) shows an absorption peak of —NH— at 3420 cm^−1^. Compared with PBA, the ‐NH‐ absorption peak of poly(PBA*x*‐*ran*‐PDMS) was red‐shifted, indicating that hydrogen bonds were formed between poly(PBA*x*‐*ran*‐PDMS) molecules.^[^ ^13^ ^]^

To investigate the mechanical properties of the structure at room temperature, we tensile tested poly(PBA*x*‐*ran*‐PDMS) (*x* = 3:1, 2:1, 1:1, 1:2 and 1:3) copolymers. As depicted in Figure 1b, with increasing PDMS content, the tensile strength of poly(PBA*x*‐*ran*‐PDMS) copolymers significantly improved from 0.01 to 0.16 MPa, whereas the elongation at break strength decreased from 4000% to 200%. The corresponding elastic modulus increased with the PDMS content. Because of the high density of intermolecular H‐bonding, strong cross‐linking, and an abundance of polar functional groups and electrostatic interactions between ‐NH^+^ and ‐OH, poly(PBA*x*‐*ran*‐PDMS) exhibits strong adhesion upon fracture contact.^[^ ^13^ ^]^ The adhesion force reached 1.7 N at a 1:1 molar ratio of PBA to PDMS (Figure 1c); hence, copolymer stiffness increases and adhesion decreases with increasing PDMS content.

Figure 1d illustrates the self‐adhesion and strength mechanism of polymers with different ratios of PBA and PDMS. PDMS facilitates strong copolymer cross‐linking by double bonding at both ends. PBA segments at the fracture self‐assemble polymer molecular chains through molecular chain motion and intermolecular reversible H‐bonding. When the content of PBA is high, fewer copolymer molecules are cross‐linked, and the mechanical stability and bearing capacity of the molecules are poor, resulting in relatively weak polymeric adhesion. A high cross‐linking effect was observed between PBA and PDMS molecules at a moderate PBA to PDMS ratio_._ Therefore, the mechanical properties and self‐adhesive force of the copolymer material were significantly improved. However, the copolymer had a higher degree of cross‐linking and a low level of molecular H‐bonding at a high PDMS content, which ultimately led to greater mechanical strength and significantly lower elongation and adhesion of the material. Therefore, optimizing the ratio of PBA to PDMS and realizing a balance between strong cross‐linking and molecular free‐chain interaction are crucial to synthesizing highly elastic poly(PBA*x*‐*ran*‐PDMS).

The macroscopic adhesive properties of the copolymer indicate that poly(PBA‐*ran*‐PDMS) can spontaneously adhere to the surfaces of various substrates, including plastic, glass, metal, PTFE, and steel (Figure S3, Supporting Information). The strong adhesion is mainly explained in two ways.^[^ ^14^ ^]^ First, the introduction of flexible PDMS chains can reduce the glass‐transition temperature of the copolymer, and its own O atoms act as H‐bonding acceptors to create strong H‐bonds in the poly(PBA‐*ran*‐PDMS) matrix, both of which contribute to the higher cohesion energy and stronger interfacial adhesion of poly(PBA‐*ran*‐PDMS). Second, in terms of chemical bonding, ‐NH‐ in PBA can form H‐bonds with the surface of O‐containing metals or N‐containing polymers (acid base interaction) and can form indirect H‐bonds with the help of humidity in space (water next to hydrophobic interface) (Figure 1e). In other words, owing to the dynamic reversibility of their non‐covalent weak interactions, supramolecular copolymers have distinct properties compared with traditional polymers, including environmental responsiveness, high deformation rates, and strong adhesiveness. However, supramolecular polymers have relatively weak intermolecular interactions and low mechanical strengths. Therefore, the development of polymer composites with high strengths and adhesion properties is of significance for expanding applications.

### 2.2 Characterizing the Structure and Mechanical Properties of Poly(PBA*x*‐*ran*‐PDMS)/VAFG

**Figure 2:**
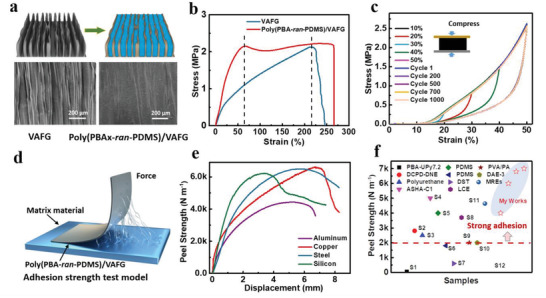
a) Synthesis process of poly(PBA*x*‐*ran*‐PDMS)/VAFG by physical impregnation. Surface micro‐topography of VAFG and poly(PBA*x*‐*ran*‐PDMS)/VAFG. b) Stress–strain curves of poly(PBA‐*ran*‐PDMS)/VAFG and VAFG; stretching speed: 5 mm min^−1^. c) Stress–strain curves and cyclic stress–strain curves (strain of 50% for 1000 cycles) of poly(PBA‐*ran*‐PDMS)/VAFG samples at different compression strains (10–50%). d) The adhesion test model of poly(PBA‐*ran*‐PDMS)/VAFG. e) Peel strength of poly(PBA‐*ran*‐PDMS)/VAFG to various substrates (aluminum, copper, steel, and silicon). f) Comparing the peel strengths of poly(PBA‐*ran*‐PDMS)/VAFG and other materials in relevant studies.

Graphene has strong mechanical properties, and filled polymers can significantly enhance its processing performance. However, conventional graphene‐based materials have poor structural continuity and high interfacial thermal resistance, and the resulting composites are prone to hole formation, which ultimately leads to lower mechanical and ***k*** values for the composite.^[^ ^15^ ^]^ To improve mechanical properties and material continuity, the polymer was filled into VAFG by the dipping‐pressure filling method (*m*_VAFG_ = 2.17 wt%). The micro‐morphology of the parallel and horizontal sections of the resulting VAFG and poly(PBA*x*‐*ran*‐PDMS)/VAFG after liquid‐nitrogen quenching was characterized (Figure S4, Supporting Information and **Figure** **2**a). VAFG has vertically aligned parallel surfaces and a uniform distribution of folds. When the polymer is filled between the folds of graphene, the surface becomes dense and uniform, and VAFG is distributed in gully shape in the cross section of Poly(PBA*x*‐*ran*‐PDMS)/VAFG, which indicates that the composite is compact. Moreover, the poly(PBA*x*‐*ran*‐PDMS)/VAFG elastomers exhibit good thermostabilities with no weight loss up to 250 °C (Figure S5, Supporting Information).

The ratio of H‐bonds in the poly(PBA*x*‐*ran*‐PDMS) derivatives plays a significant role in imparting mechanical strength and other properties with different cross‐links at the interface^22^. Therefore, the effect of the PBA to PDMS molar ratio on the mechanical strength of the poly(PBA*x*‐*ran*‐PDMS)/VAFG was investigated. In Figure S6a,b, Supporting Information, following the same trend as Figure 1b, the maximum tensile strength of the composite was observed to significantly increase with the PDMS ratio. However, the elongation of the composite also significantly decreased owing to the degree of cross‐linking. In particular, the strength of poly(PBA‐*ran*‐PDMS) was 0.2 ± 0.05 MPa and its elongation dropped to 1800% at a 1:1 PBA to PDMS ratio. For VAFG and the composite (Figure 2b), the strengths of VAFG and poly(PBA‐*ran*‐PDMS)/VAFG reached 2.02 ± 0.05 and 2.23 ± 0.15 MPa, respectively, while the elongations of VAFG and poly(PBA‐*ran*‐PDMS)/VAFG reached 250% and 270%, respectively. VAFG eventually broke, and the mechanism was further corroborated by the decrease in breaking elongation with increasing strain rate, which indicates that a higher proportion of PDMS leads to higher strength and lower elongation. Therefore, the exceptional stretchability and mechanical strength of the resulting network are attributable to two key aspects of the proposed mechanism:^[^ ^16^ ^]^ i) The presence of H‐bonds causes the network to stretch by a hierarchical energy dissipation mechanism; ii) the dry network bears a large number of high‐density cross‐linking sites, leading to highly folded polymer chains that facilitate easier chain sliding owing to a decrease in the interchain distances. Furthermore, poly(PBA‐*ran*‐PDMS)/VAFG exhibits good resilience at 10–50% compression strain and maintains 1000 cycles at 50% strain (Figure 2c). The excellent elastic deformation and interfacial adhesion performance of poly(PBA‐*ran*‐PDMS)/VAFG not only fill microgaps and rough surface holes and other interfaces with complex surface structures, but also maintains close contact during the thermal expansion and contraction of the material.^[^ ^17^ ^]^

In addition, the peel strength of poly(PBA‐*ran*‐PDMS)/VAFG on different substrates was tested. Figure 2d illustrates a schematic showing adhesion testing of poly(PBA‐*ran*‐PDMS)/VAFG. Interestingly, compared with its Al and Si counterparts, poly(PBA‐*ran*‐PDMS)/VAFG exhibited a higher adhesion strength on the surfaces of stainless steel (6320 N m^−1^) and Cu (6500 N m^−1^), which is attributable to the —OH groups and the oxide layer on the metal surface (Figure 2e). Poly(PBA‐*ran*‐PDMS)/VAFG exhibited better adhesive peel strength than the adhesive elastomer reported in the literature (Figure 2f, Table S2, Supporting Information). Higher interfacial adhesion and elasticity improve interfacial interactions between heterogeneous materials and promote the heat transfer efficiency and sensing performance of the material.

### 2.3 Heat Transfer Simulation and Performance Testing of Poly(PBA*x*‐*ran*‐PDMS)/VAFG

**Figure 3:**
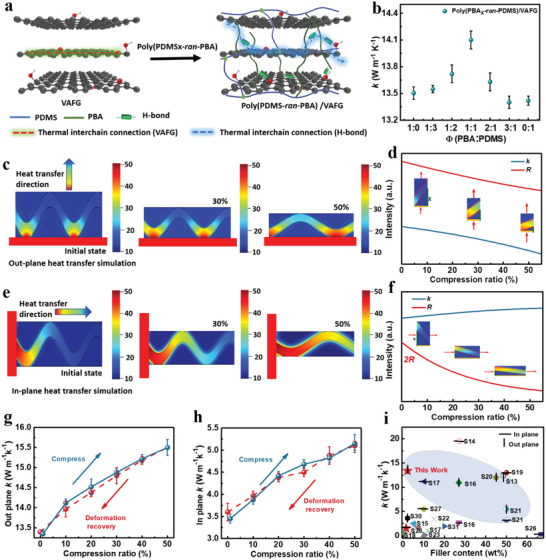
*k* values of amorphous polymers and their VAFG blends. a) Schematic of the heat transfer paths of VAFG and poly(PBA‐*ran*‐PDMS)/VAFG. The *k* values of b) poly(PBA*x*‐*ran*‐PDMS)/VAFG with different molar ratios (*x* = 1:0, 1:3, 1:2, 1:1, 2:1, 3:1, 0:1). The error line is determined based on the thickness, size, and results of multiple film testing. Simulation of c,d) out‐plane and e,f) in‐plane heat transfer performance of poly(PBA‐*ran*‐PDMS)/VAFG at different pressures using COMSOL Multiphysics. g) In‐plane and h) out‐plane *k* values of poly(PBA‐*ran*‐PDMS)/VAFG at different compression rebound values. i) Comparing *k* for various filler contents in the literature.

VAFG reduces the distance and time of phononic transmission, improving the directional ***k*** values of composites (**Figure** **3**a). The results indicate the important contributions of energy diffusion through non‐propagating vibrational modes, the anharmonic coupling of localized modes, and the ballistic propagation of delocalized modes to heat transfer.^[^ ^8^, ^18^ ^]^ In polymeric systems, heat is transferred significantly more efficiently along a strong bonded chain than between chains bonded by weak interactions. The H‐bonding in these systems is ≈10–100 times stronger than the van der Waals interactions. H‐bonds have sufficient design flexibility, and their directionality and intermolecular interactions make them another contributor to the ***k*** value of the polymer.^[^ ^19^ ^]^ Therefore, increasing the strength and intermolecular interactions of the molecular bonds generally improves the ***k*** value of polymers.

To realize a uniform and homogeneous distribution of H‐bonds, the H‐bond‐accepting and ‐donating polymers are required to not only be homogeneously dispersed but also to be miscible at the molecular level to allow polymers to intertwine within the radius of gyration. Figure S7, Supporting Information shows the test results of the ***k*** value of poly(PBA*x*‐*ran*‐PDMS) at different PDMS‐to‐PBA ratios (*x* = 1: 0, 1: 3, 1: 2, 1: 1, 2: 1, 3: 1, 0: 1). The ***k*** value first increases and then decreases with increasing PDMS content. The highest ***k*** value of poly(PBA*x*‐*ran*‐PDMS) occurs at *x* = 1:1 (0.27 ± 0.025 W m^−1^ K^−1^), which indicates that two strong H‐bonds are formed between PBA molecules for thermal linkage between chain segments, whereas a high concentration of PDMS leverages its main chain rigidity to promote the extended conformations of strong hydrogen bonds.^[^ ^13d^ ^]^ When the percolation threshold is exceeded, the concentration of PDMS disrupts the uniform distribution of strong H‐bonds, reducing the ***k*** value of the material. Therefore, optimizing the PBA and PDMS contents is important for studying the ***k*** value of the material, which is significantly higher at *x* = 1:1 (13.4 ± 0.3 W m^−1^ K^−1^) (Figure 3b). Similarly, the ***k*** value of poly(PBA*x*‐*ran*‐PDMS)/VAFG composites increases and then decreases with the PDMS content. This indicates that, in addition to the effects of polymeric interactions, interfacial interactions between the polymer and graphene increase and then decrease by polymer adhesion.^[^ ^20^ ^]^ High interfacial adhesion reduces the interfacial thermal resistance inside the composite and improves the ***k*** value of the material.

To understand the heat transfer effect of the material, heat transfer simulations were conducted with poly(PBA‐*ran*‐PDMS)/VAFG at different compressive deformations. The thermal simulation model is designed based on the belt‐shaped sinusoidal scaffold of VAFG in the poly(PBA*x*‐*ran*‐PDMS)/VAFG composites according to similar models in previous studies.^[^ ^21^ ^]^ The simulations reveal a distinct increase in the heat transfer rate of the interfacial material after compression. The results show that the interfacial thermal resistance of the material decreases and the ***k*** value increases with increasing compression ratio for the same heat transfer time (Figure 3c). In addition, we assumed that the graphene structure was stable, the thermal resistance of graphene and polymer was insignificant, and the cell length was constant under all compression conditions. With the increase in the compression ratio, the trends in ***k*** and interfacial thermal resistance were in accordance with Equations (1) and (2), respectively.

(1) $$k=\frac{k_{i}x}{2\sqrt{x^{2}+1}}$$

(2) $$R=2R_{i}\sqrt{x^{2}+1}$$

where ***k*_i_** is the ***k*** value of graphene, *R*_i_ is the graphene interfacial thermal resistance, and *x* is the compression ratio. In Figure 3d, graphene and the material substrate tend to be horizontal with increasing compression ratio, the out‐plane ***k*** value gradually decreases, and the effective interfacial thermal resistance reaches its maximum. Moreover, the curvature of compression gradually flattens, and the heat transfer efficiency in the in‐plane direction is significantly improved with the compression ratio (Figure 3e). Similarly, the microelements of unit length were analyzed assuming constant volume and elongation of the cell length with increasing compression ratio, with the trends of ***k*** and interfacial thermal resistance found to be in accordance with the following equations:

(3) $$k=\frac{k_{i}V}{x\sqrt{x^{2}+1}}$$

(4) $$R=\frac{2R_{i}x}{\sqrt{x^{2}+1}}$$

A plot of the two equations reveals that the effective thermal resistance in the horizontal direction gradually decreases, and the ***k*** value gradually increases to its maximum with increasing length after compression (Figure 3f). In addition, the out‐plane ***k*** values of poly(PBA‐*ran*‐PDMS)/VAFG were tested at different compression strengths. The results demonstrate that the initial out‐plane ***k*** value of the material was 13.4 ± 0.3 W m^−1^ K^−1^, which is in proportion to the compression ratio. The interfacial thermal resistance is inversely proportional to the compression ratio. The out‐plane and in‐plane ***k*** values were 15.49 ± 0.5 W and 5.30 ± 0.3 W m^−1^ K^−1^, respectively, when compressed to 50% (Figure 3g,h). The in‐plane or out‐plane thermal conductivity of the composite increases significantly. And the influence of thermal conductivity of composites under different compression can be divided into three aspects. 1) After compression, the density of VAFG within the composite increases and the heat transfer efficiency of the material improves. 2) After compression, the interfacial contact between VAFG and between VAFG and polymer is enhanced, phonon scattering and interfacial thermal resistance are reduced, and thermal conductivity is improved. 3) Theoretically, with the increase of compression ratio, the density of the composite increases, the interfacial contact effect is enhanced, and the thermal conductivity increases. However, as the compression ratio of the composite increases and the horizontal direction graphene materials are arranged in parallel, the out‐plane k increases, and the in‐plane k decreases. Because the compression ratio of poly(PBA‐*ran*‐PDMS)/VAFG composite is 0–50%, the orientation structure of the material was not damaged and the interfacial contact effect was enhanced during the compression process. Therefore, as the compression ratio increases, the density of the composite increases in the horizontal direction, the relative heat transfer path in the vertical direction decreases, and the in‐plane or out‐plane thermal conductivity of the composite increases significantly.

In addition, after slow release, the ***k*** values of the materials returned to those of their initial states. In addition, among the comparable materials reported in the literature, poly(PBA‐*ran*‐PDMS)/VAFG has the lowest graphene content and a higher ***k*** value relative to in‐plane and out‐of‐plane values (Figure 3i, Table S3, Supporting Information), which confirms that the process plays a crucial role in improving the ***k*** value of the material.

### 2.4 Interfacial Heat Transfer Simulation of Poly(PBA‐*ran*‐PDMS)/VAFG

**Figure 4:**
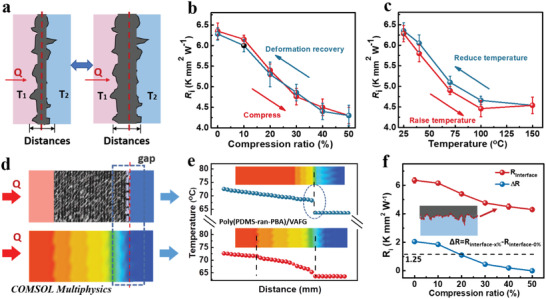
a) Model of heat transfer at the interface of a poly(PBA‐*ran*‐PDMS)/VAFG material. Interfacial thermal resistance curves between poly(PBA‐*ran*‐PDMS)/VAFG and Cu b) at different compression forces and c) temperatures. d) Steady‐state temperature profile for the heat transfer model under compression from COMSOL Multiphysics thermal simulations. e) Heat flows across the junction of the models with or without poly(PBA‐*ran*‐PDMS)/VAFG interfacial materials from source to sink under compression.

The rapid directional thermal evacuation at the junction of two types of material with a large temperature difference is key for maintaining the temperature balance of the structural system.^[^ ^22^ ^]^ Highly thermally conductive elastomers fill the irregular pores of the interface, improve phonon transfer efficiency at the interface, reduce air thermal radiation, realize rapid heat transfer, and maintain good thermal contact with the interface in the cyclic variable load environment (**Figure** **4**a).^[^ ^23^ ^]^ Moreover, they prevent weak contact or interfacial detachment, owing to the difference in thermal expansion and contraction between the two sides of the material, and reduce contact thermal resistance.

To understand the heat transfer effect of poly(PBA‐*ran*‐PDMS)/VAFG, it was subjected to interfacial thermal resistance testing at different compressive deformations and temperatures. The results demonstrate that the ***k*** value is proportional to the compression ratio, whereas the interfacial thermal resistance is inversely proportional to the compression ratio. The initial interfacial thermal resistance of poly(PBA‐*ran*‐PDMS)/VAFG and Cu was 6.35 ± 0.2 K mm^2^ W^−1^. When compressed by 50%, the interfacial thermal resistance became 4.30 ± 0.2 K mm^2^ W^−1^ (Figure 4b). After slow release, the interfacial thermal resistance of the material returned to its initial value, indicating that the ***k*** value of the material is stable in its various morphologies. In addition, the interfacial thermal resistance was tested at different temperatures, and the results show that it decreases in the 25–100 °C range and plateaus in the 100–150 °C range (Figure 4c). This is because the adhesion and interfacial bonding of molecules increase with temperature, whereas the adhesion of the polymer matrix tends to stabilize at high temperatures.

However, when traditional polymer interfacial materials are connected to a metal heat source, rapid directional thermal evacuation with a large temperature difference is the key to maintaining the temperature balance at the connection of the structural systems (heat source → heat sink) (Figure 4d). To understand the mechanism for thermal conductance enhancement, we performed a detailed atomistic level study using COMSOL multiphysics thermal simulation.^[^ ^24^ ^]^ Compared with traditional heat source/radiator junctions, the poly(PBA‐*ran*‐PDMS)/VAFG layer enhances the thermal conductance of poly(PBA‐*ran*‐PDMS)/VAFG/Cu interfaces (Figure 4e). Moreover, the total comprehensive thermally conductive and interfacial thermal resistance of poly(PBA‐*ran*‐PDMS)/VAFG‐Cu with different compressions and rebound ratios were tested (Figure 4f). The ***k*** value of poly(PBA‐*ran*‐PDMS)/VAFG‐Cu increased and the interfacial thermal resistance decreased with the increasing compression ratio. In the initial state, the interfacial thermal resistance of poly(PBA‐*ran*‐PDMS)/VAFG and Cu was 6.35 ± 0.2 K mm^2^ W^−1^, while it was 4.3 ± 0.1 K mm^2^ W^−1^ at 50% compression. The changed thermal resistance of the interface between the material and the substrate remained ≈1.25 ± 0.01 K mm^2^ W^−1^ before and after compression, which indicates that poly(PBA‐*ran*‐PDMS)/VAFG exhibits good interfacial adaptability. Therefore, poly(PBA‐*ran*‐PDMS)/VAFG with high ***k*** and elasticity as well as good adhesion can fill the irregular interface, reduce the interfacial spacing, and improve the interfacial interaction force and phonon transfer efficiency, making it the best thermal conductor.^[^ ^25^ ^]^

The addition of VAFG not only helps to improve the mechanical and thermal properties of poly(PBA‐*ran*‐PDMS)/VAFG, but also endows poly(PBA‐*ran*‐PDMS)/VAFG with sensitive strain conductivity. During stretching, compression, or temperature testing, resistance instantly responds to changes in the deformation or temperature of the poly(PBA‐*ran*‐PDMS)/VAFG sensor, thereby demonstrating fast sensitivity (Figure S8a–c, Supporting Information)).^[^ ^19^ ^]^ Subsequently, joint‐motion was monitored by integrating poly(PBA‐*ran*‐PDMS)/VAFG on the finger of the manipulator (Figure S8d, Supporting Information). The resistance of poly(PBA‐*ran*‐PDMS)/VAFG increases differently as the finger is bent to different angles (0°, 30°, 45°, and 90°). The degree of bending is accurately reflected using the degree of resistance change. Hence, the poly(PBA‐*ran*‐PDMS)/VAFG sensor can be used to detect the bending motion of a finger. In addition, the corresponding resistance of poly(PBA‐*ran*‐PDMS)/VAFG exhibited repeatable signal changes when the finger was bent at 30°, 60°, and 90°, indicating that a poly(PBA‐*ran*‐PDMS)/VAFG sensor will be very stable and capable of long‐term deformed‐object monitoring (Figure S8e,f, Supporting Information)). Poly(PBA‐*ran*‐PDMS)/VAFG sensors improve the accuracy and feasibility of pressure signals, and realize the real‐time monitoring of temperature and pressure signals.

### 2.5 Manipulator Learning Perception Based on Poly(PBA‐*ran*‐PDMS)/VAFG

**Figure 5:**
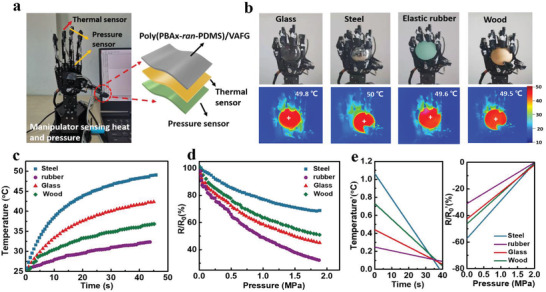
Applications of materials. a) Manipulator model and sensor structure design. b) Gripping balls of different materials (glass, stainless steel, elastic rubber, and wood). Infrared thermal camera images of the manipulator grasping different objects simultaneously. c) Temperature changes at different times after the manipulator has gripped the four materials. d) Resistance changes at different pressures during gripping. e) Trend fitting of temperature–time curve and *R*/*R*_0_–pressure curve.

Poly(PBA‐*ran*‐PDMS)/VAFG has excellent interfacial adhesion, high elastic deformation, a high ***k*** value, and rapid resonance reflection under different pressure and temperature signals. As temperature and hardness are crucial for analyzing and identifying the basic physical characteristics of objects, poly(PBA‐*ran*‐PDMS)/VAFG materials are promising candidates for applications in bionic tactile perception and recognition. To explore the sensory properties of the material, it was mounted on a manipulator. **Figure** **5**a illustrates the model of a flexible tactile sensor applied to a manipulator. The fingers of the manipulator were assembled in the order of poly(PBA‐*ran*‐PDMS)/VAFG material > thermal sensor > pressure sensor. Owing to the elasticity and adhesion of the material, the manipulator easily grasped objects (balls) with different hardnesses, roughnesses, and ***k*** values. Figure 5b shows effect plots of the manipulator grasping balls with different materials and hardnesses at the same temperature. In the initial state, the four grasping effect plots were examined at room temperature by infrared thermal imaging. The results demonstrate that the temperatures of stainless steel, glass, rubber, and wood are the same in the initial state.

To determine the potential of the gripper in sensing physical properties such as temperature and pressure, the manipulator was tested using temperature–time and pressure–resistance signals while gripping different types of balls. Figure 5c illustrates temperature variations of the manipulator gripping glass, stainless steel, rubber, and wood at different times. We found that the temperature of the manipulator when grasping different balls simultaneously followed the order: *T*_stainless steel_ > *T*_glass_ > *T*_rubber_ > *T*_wood_. This indicates that the higher ***k*** value of the ball, the higher the temperature perceived by the manipulator after grasping, at the same transfer time. Figure 5d shows curves of pressure and electrical signals perceived by the fingers of the manipulator as it grabs the balls. We also found that changes in the electrical signals perceived by the manipulator after grasping the four types of ball followed the order: *R*/*R*_0rubber_ < *R*/*R*_0glass_ < *R*/*R*_0wood_ < *R*/*R*_0stainless steel_, which indicates that, at the same stress, the greater the deformation of the material, the faster the change in resistance of the manipulator during gripping and the more sensitive the electrical signals. To further understand these trend curves, temperature–time (*T*–*s*) and *R*/*R*_0_–pressure curves (*R*/*R*_0_–*P*) were constructed. The *T*–*s* (25–50 °C) and *R*/*R*_0_–*P* curves each of the four spheres were fitted to Equation (5), the result of which are summarized in Table S4, Supporting Information.

(5) $$y=Ax^{2}+Bx+C$$

The derivative of the fitting curve was derived over the test range, and the change trends of the temperature and electrical signals of the manipulator after grasping different types of balls were visually reverified, which revealed that the manipulator with the poly(PBA‐*ran*‐PDMS)/VAFG material with good adhesion, elasticity, and a high ***k*** value can efficiently sense temperature and pressure changes upon grasping different objects, which is of great significance for the perception and recognition of unknown materials.^[^ ^26^ ^]^

### 2.6 Perception and Recognition of the Manipulator Based on Poly(PBA‐*ran*‐PDMS)/VAFG

**Figure 6:**
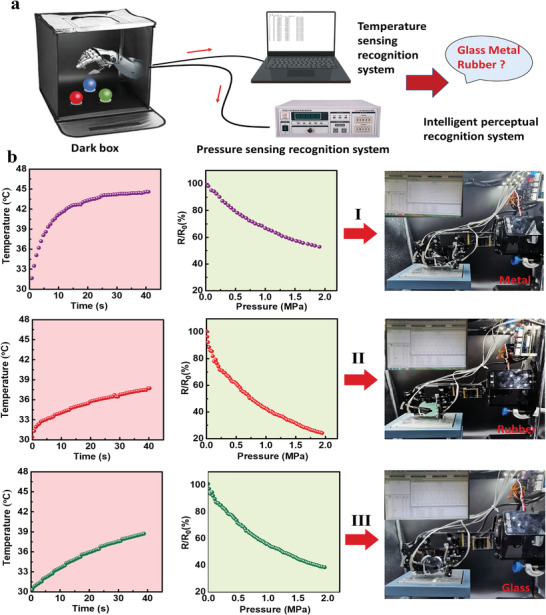
Perception and recognition of materials in a dark box. a) Design model for the capture of three unknown substances in a dark box. b) Temperature–time curves and pressure–electric signal curves after grabbing three unknown substances in a dark box.

Smart materials emulate perception as a prerequisite to realizing intelligent application in sensors. The identification of unknown materials is a major goal of intelligent material applications.^[^ ^27^ ^]^ As the manipulator equipped with poly(PBA‐*ran*‐PDMS)/VAFG sensors exhibits significantly different heat transfer and pressure trends with different objects, an object made of unknown materials can be identified and tested using the manipulator in a dark box. **Figure** **6**a depicts the schematic of the test system, where the material is heated to a constant temperature in a dark box, after which the manipulator grabs the ball, reads the temperature change and pressure change data simultaneously, and plots and analyses the data. The trend is then compared with a database of known perceptions to obtain the material texture of the ball.

Figure 6b shows *T*–*s* and *R*/*R*_0_–*P* curves obtained for previously tested metal, glass, and rubber balls grasped randomly by the manipulator in a dark box. According to the temperature curve trend, the temperature rate is higher when the manipulator grasps ball **I**, and sensing changes are slow. This indicates that the material has high heat transfer efficiency and hardness, and the curve slope is consistent with that of a metal ball; ball **I** is a metal ball. As the manipulator grips ball **II**, the temperature rises sluggishly under the same conditions, but the pressure sensing signal changes strongly. The trend is consistent with the test results of the rubber ball; therefore, ball **II** is a rubber ball. Using the same principle, we identified ball **III** to be a glass ball. The sensing signals from the three types of balls were further compared and found to be consistent with the trend: *R*/*R*_0rubber_ < *R*/*R*_0glass_ < *R*/*R*_0stainless steel_. Finally, the accuracy of the results was reverified by combining photographic images of the test states. The results indicate that the poly(PBA‐*ran*‐PDMS)/VAFG material has the potential to realize perception and recognition in sensors and has promising development prospects in terms of capture and operability. Poly(PBA‐*ran*‐PDMS)/VAFG with high adhesion, high ***k*** value, and low interfacial thermal resistance has immense applications prospects in sensing, such as information recognition and self‐learning.

## 3 Conclusions

In summary, high adhesion and elongation poly(PBA‐*ran*‐PDMS) polymers were synthesized by optimizing supermolecular interactions and suitable cross‐linked chains. Adhesive composites with high elasticities and thermal conductivities were also synthesized by bonding VAFG with poly(PBA‐*ran*‐PDMS) copolymer. The out‐of‐plane ***k*** value of the material reached 15.49 ± 0.5 W m^−1^ K^−1^, the in‐plane ***k*** value reached 5.30 ± 0.3 W m^−1^ K^−1^, and the interfacial thermal resistance between materials and Cu was 6.35 ± 0.2 K mm^2^ W^−1^. In addition, the material exhibits robust sensing performance as resistance significantly changes at different applied pressures. In application, poly(PBA‐*ran*‐PDMS)/VAFG‐containing manipulators precisely captured, sensed, and recognized various materials. Thus, this study established a method for designing polymer‐based composites with complex functional thermal, sensing, and mechanical conductivity integration, which provides a theoretical basis and technical support for the design and preparation of future high‐performance polymer‐based interfacial materials; it also provides broad potential prospects for the development of soft robots and bionic prosthetics.

## 4 Experimental Section

### Materials

Poly‐2‐[[(butylamino)carbonyl]oxy]ethyl ester (PBA), Polydimethylsiloxane, vinylendblocked (PDMS), ethyl acetate, 2,2'‐azobis(2‐methylpropionitrile), and dimethylacetamide (DMAC, super dry) were purchased from Seans Co., Ltd. GO was synthesized by an improved Hummers method.^[^ ^28^ ^]^ The graphene film has a thermal conductivity of 700 W m^−1^ K^−1^, a density of 1.18 g cm^−3^, and a specific heat of 0.735 J g^−1^ K^−1^. The vertically aligned folded graphene (VAFG) was prepared by the template heat shrink method. Deionized water was prepared in the laboratory.

### Synthesis of 2‐[[(Butylamino)Carbonyl]Oxy]Ethyl Ester–Vinyl‐End‐Terminated Polydimethylsiloxane (Poly(PBA*x*‐*ran*‐PDMS))

Different molar ratios of PBA and PDMS (PBA: PDMS = 1: 1, 1: 2, 1: 3, 3: 1, 2: 1) were added to 20 mL of DMAC and stirred at room temperature for 2 h. 2, 2'‐Azobis(2‐methylpropionitrile) (0.1 mmol, 16.41 mg) was added to this mixture and reacted at 68 °C for 24 h (Ar). The reaction product was precipitated in deionized water, and the resulting precipitate was dissolved in ethyl acetate.

### Preparation of Reduced Graphene Membranes

First, 20 mL of 2 mg mL^−1^ aqueous graphene oxide solution was extracted into graphene oxide membranes by using the extraction method. Then, the preliminarily reduced graphene film was fumigated with HI acid at 60 °C in a confined space for 2 h. Then, the graphene film was heated at 500 °C in the mixed atmosphere of 500 sccm Ar and 50 sccm H_2_ for 10 min to obtain the reduced graphene film. Then, the graphene film was obtained by hot pressing at 300 °C for 2 h.

### Preparation of Vertically Aligned Folded Graphene (VAFG)

First, the tape with a thickness of 2 mm was stretched in one direction to 400 times its original length. Then, the graphene film was adhered to the surface of the tape, and the tape was slowly released to the initial length. Then, the material was immersed in ethanol solvent for 2 h. The tape was separated from the graphene membrane, and vacuumed at 80 °C for 6 h to obtain VAFG.

### Synthesis of Poly(PBA*x*‐*ran*‐PDMS)/VAFG

The poly(PBA*x*‐*ran*‐PDMS) ethyl acetate solution was filled into VAFG by physical impregnation, dried at 25 °C for 12 h, and then vacuum dried for 24 h. The poly(PBA*x*‐*ran*‐PDMS)/VAFG composite was obtained by repeating this process several times until fully filled.

### Structural Analysis and Characterization

The morphologies and microstructures of the poly(PBA*x*‐*ran*‐PDMS) and poly(PBA*x*‐*ran*‐PDMS)/VAFG composite materials were characterized by field‐emission scanning electron microscopy (FESEM; S4800‐15 kV, Hitachi, Japan). The chemical structures of copolymer samples were examined by Fourier‐transform infrared (FT‐IR) spectroscopy (Tensor27, Bruker, Germany). The thermal stabilities and glass transition temperatures (***T***_g_) of the samples were determined by thermal gravimetric analysis (TGA; Q5000, TA Instruments, Japan) and differential scanning calorimetry (DSC; TA660, TA Instruments, Japan), respectively (10 °C min^−1^ in Ar). ^1^H Nuclear magnetic resonance (^1^H‐NMR) spectra were acquired on a 400 MHz spectrometer (NMR; AVANCE III, Bruker, Swiss). The ***k*** values of poly(PBA‐*ran*‐PDMS)/VAFG and the heat transfer interface were investigated by COMSOL Multiphysics 5.4. The values of the parameters used for the ed system are listed in Table S1, Supporting Information. The mass ratio of VAFG (***m***_VAFG_) to the total mass (***m***) of composite was calculated using Equation (6):

(6) $$\Delta M=\frac{m_{\mathrm{FGf}}}{m}\times 100\%$$

### Mechanical Properties

All mechanical tensile properties were examined using a universal mechanical testing machine (XQ‐1C, XinXian, China) using samples 35.0 × 12.8 × 0.25 mm in size at a tensile rate of 5 mm min^−1^. Moreover, unconfined compression testing was carried out (Φ12 × 10 mm). Additionally, the adhesion area of the material was 200 mm^2^ and the stretching speed was 5 mm min^−1^.

### Electrical Response

Electrical responses were measured at different temperatures, compressions, and tensile deformations using a TH2830 LCR meter and an electrochemical workstation (CHI660E, Chenhua, China). Part of the *R*_0_/*R* output current data (*I*) was converted by the electrochemical workstation directly into the corresponding current signal (*I*/*I*_0_) (*I*/*I*_0_ = *R*_0_/*R*) according to Ohm's law.

### Thermal Conductivity

The thermal transport properties of the samples were visually recorded by the infrared thermal imager (TiX 660, Fluke, USA). First, the balls (Φ30 mm) of different materials (glass, stainless steel, elastic rubber, and wood) were placed in an air‐circulating oven at 50 °C to ensure a uniform surface temperature dispersion. Then, the balls were grasped using a manipulator at room temperature. At the same time, the state of the gripped ball was characterized using an infrared thermal imager. The infrared thermal imager was 30 cm away from the object, and the storage time was ≈10 s. The environmental condition was natural light.

The thermal transport properties and thermal diffusivities of the samples (Φ10 × 3 mm) were also measured by the heat flow method (Xiangtan Xiangke, DRL‐2A, China). The in‐plane thermal conductivity was tested using laser thermal conductivity meter (LFA467, NETZSCH, Germany). The temperature of the hot pole was 60 °C and the temperature of the cold pole was 25 °C during the test. According to the compression ratio, the thickness of the material was adjusted using a self‐made mold. Then the thermal conductivity of poly(PBA‐*ran*‐PDMS)/VAFG was tested under different compression. It should be noted that the test process should ensure that the surface of samples was in full contact with the test probe. The interfacial thermal resistance (***R***, K mm^2^ W^−1^) of each sample was calculated using the following equations:

(7) R=d/k

where ***k*** is the thermal conductivity (W m^−1^ K^−1^), and ***d*** is the thickness of thermal interfacial material (m).

## Conflict of Interest

The authors declare no conflict of interest.

## Supporting information

Supporting Information

## Data Availability

The data that support the findings of this study are available from the corresponding author upon reasonable request.
