# Supplementary material for: Highly Thermally Conductive Adhesion Elastomer Enhanced by Vertically Aligned Folded Graphene
Source: Adv Sci (Weinh). 2022 Oct 17;9(33):2201331. doi: 10.1002/advs.202201331 (PMC9685443; doi:10.1002/advs.202201331)
Supplement: Supplementary file 1 — Supporting Information [file ADVS-9-2201331-s001.pdf]

Supporting Information

Highly thermally conductive adhesion elastomer enhanced by vertically aligned folded graphene

Huitao Yu, Yiyu Feng, Can Chen, Heng Zhang, Lianqiang Peng, Mengmeng Qin, Wei Feng**

Dr. H. Yu, Prof. Y. Feng, Dr. C. Chen, Dr. H. Zhang, Dr. L. Peng, Dr. M. Qin, Prof. W. Feng

Tianjin Key Laboratory of Composite and Functional Materials, School of Materials Science and Engineering, Tianjin University, Tianjin 300350, P. R. China

Prof. Y. Feng,

Key Laboratory of Materials Processing and Mold, Ministry of Education, Zhengzhou University, Zhengzhou 450002, P. R. China

*Corresponding author. weifeng@tju.edu.cn (W. Feng)

fengyiyu@tju.edu.cn (Y. Feng)

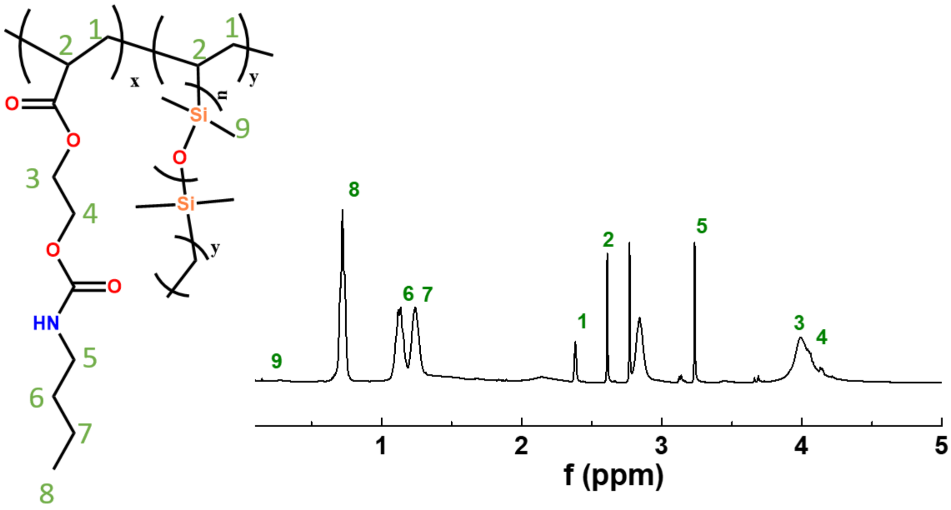

Figure S1 ^{1}H NMR spectra of poly(PBAx-*ran*-PDMS) in $CDCl_3$.

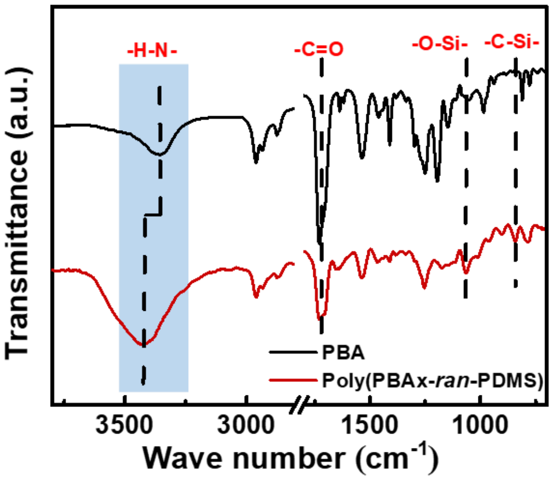

Figure S2 FTIR spectra of PBA and poly(PBAx-*ran*-PDMS).

The characteristic chemical structures of the PBA and poly(PBAx-*ran*-PDMS) were investigated through Fourier transform infrared analyses. The PBA exhibited stretching vibration peaks of C=O and C–N at 1725 and 1534 cm^{-1}, respectively. The poly(PBA-*ran*-PDMS) exhibited stretching vibration peaks of N–H and –C–Si– at 3340 and 840 cm^{-1}, respectively.

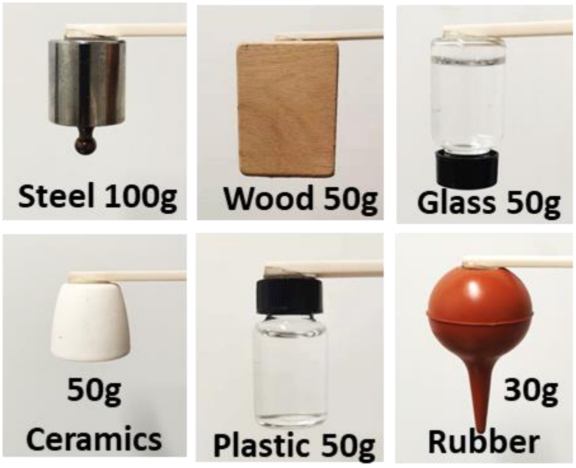

Figure S3 Photographs of the poly(PBA-*ran*-PDMS) adhering to various substrates: steel, wood, glass, ceramics, plastic and rubber.

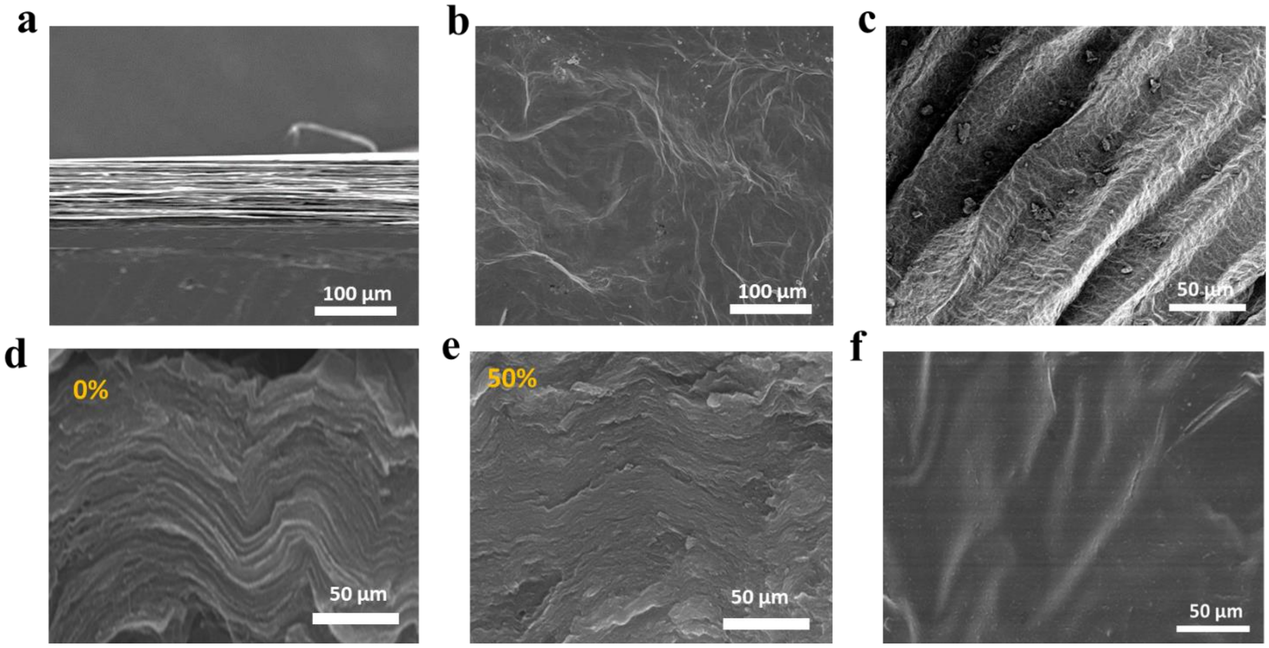

Figure S4 (a) Cross section and (b) surface morphology for graphene film. (c) Surface morphology for VAFG. Cross section morphology for (d, e) poly(PBA-*ran*-PDMS)/VAFG before and after compression. (f) surface morphology for poly(PBA-*ran*-PDMS)/VAFG film.

The microscopic morphology of graphene films, VAFG and poly(PBA-*ran*-PDMS)/VAFG composites were characterized in **Figure S4**. The results show that the graphene film has a dense structure, the lamellae are arranged in parallel, and the thickness of the film is about 100 μm (**Figure S4a**). In addition, the surface of the graphene film is smooth and no defects (**Figure S4b**). The surface morphology of VAFG is arranged and folded, with a complete morphological structure and a fold width of about 50 μm (**Figure S4c**). When the poly(PBA-*ran*-PDMS) was filled to both sides of VAFG to obtain the poly(PBA-ran-PDMS)/VAFG composite. The composite is dense and flat, and the graphene is completely encapsulated by the polymer (**Figure S4d, e**). Moreover, the polymer is filled into the gap of VAFG and the surface morphology of poly(PBA-*ran*-PDMS)/VAFG is flat, smooth and without obvious defects (**Figure S4f**). The proportion of VAFG skeleton to the total mass of composites (ΔM) was calculated as the mass of the VAFG (m_{VAFG}) divided by the total volume of the composite (m) (**Equation S1**).

$$\boldsymbol{\Delta M = \frac{m_{FGf}}{m} \times 100\%} \qquad (1)$$

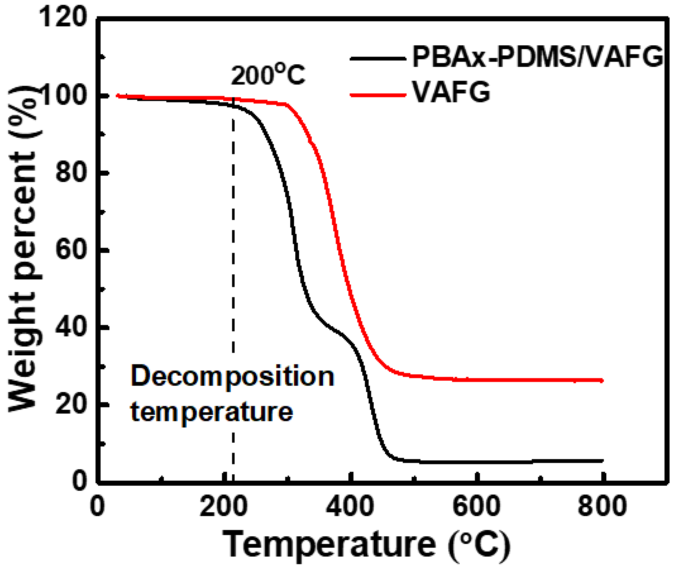

Figure S5 TG spectra of VAFG and poly(PBA-*ran*-PDMS)/VAFG.

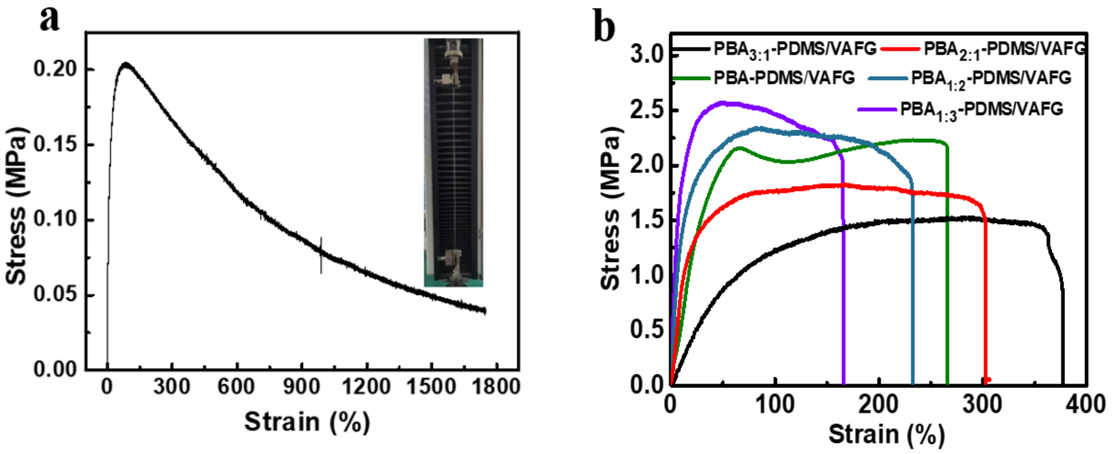

Figure S6 (a) Strain–stress curves of poly(PBA-*ran*-PDMS). The insert map is the digital photograph of the material stretching. (b) Stress-strain curves of the poly(PBAx-*ran*-PDMS)/VAFG with different molars of PBA and PDMS.

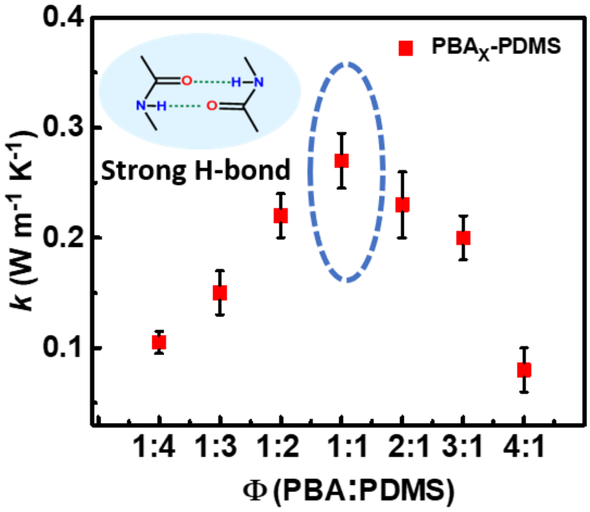

Figure S7 Thermal conductivity of poly(PBAx-*ran*-PDMS) with different molar ratios of PBA and PDMS (x=1:0, 1:3, 1:2, 1:1, 2:1, 3:1, 0:1). The error line was determined based on the thickness, size and multiple testing results of the film.

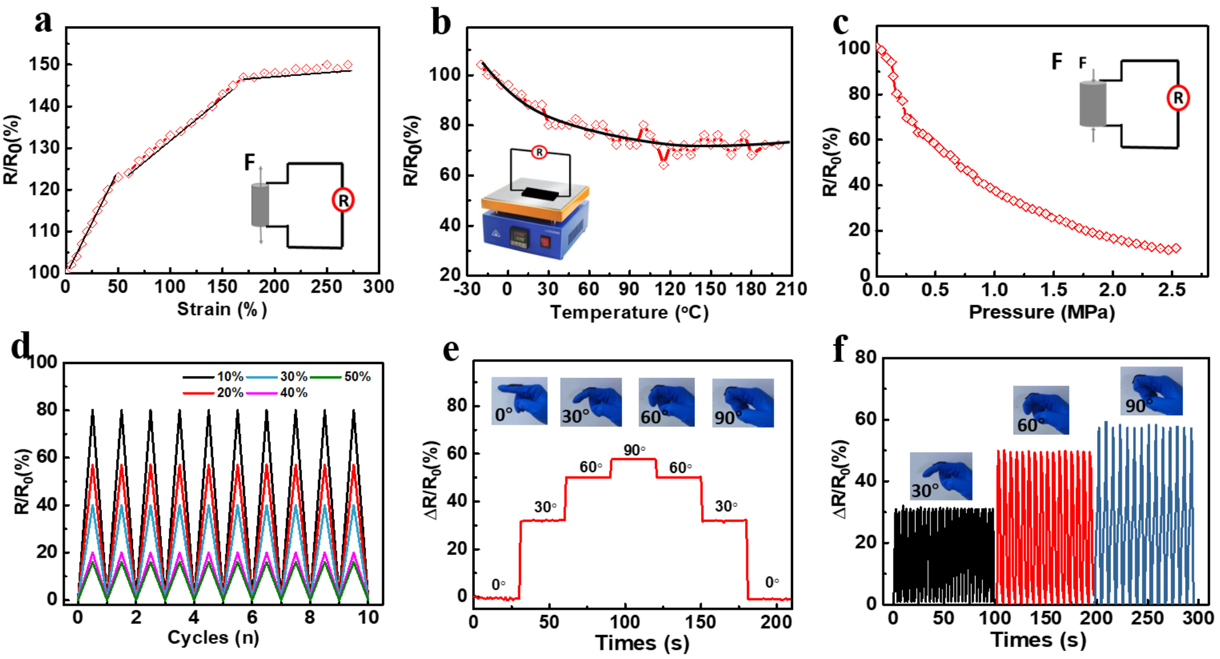

Figure S8 Performance of poly(PBA-*ran*-PDMS)/VAFG based flexible sensors. (a) Relative resistance curves of the poly(PBA-*ran*-PDMS)/VAFG during different strain. The insert shows the test model. (b) Temperature-to-resistance and (c) pressure-to-resistance responses curves of poly(PBA-*ran*-PDMS)/VAFG based sensors. The inset shows the test model. (d) Cyclic measurement of relative resistance of poly(PBA-*ran*-PDMS)/VAFG sensor under different pressures (10%-50%). (e) The enantiomeric resistance response of the poly(PBA-*ran*-PDMS)/VAFG sensor at different bending angles (0°, 30°, 60°, 90°) of the index finger (the inserts show the finger schematic). (f) The enantiomeric resistance response of poly(PBA-*ran*-PDMS)/VAFG based pressure sensors after different cycles of bending angles (30°, 60°, 90°).

The norm factor (GF) is defined as the slope of the resistance–strain curve and commonly used to evaluate the sensitivity of strain sensors. **Figure S8a** shows that the GF of poly(PBA-*ran*-PDMS)/VAFG sensor can reach about 1.1 and 0.8 when the strain is 0-50% and 50-175%, respectively. And when the strain is 175%, the resistance of poly(PBA-*ran*-PDMS)/VAFG is basically unchanged without any significant loss, indicating that the electrical properties of the poly(PBA-*ran*-PDMS)/VAFG have good stability. Notably, the poly(PBA-*ran*-PDMS)/VAFG sensor is sensitive to both pressure and temperature due to the fast ion mobility, and subsequent tests of pressure/resistance (**Figure S8b**) and temperature/resistance were tested. Because of the fold

structure of poly(PBA-*ran*-PDMS)/VAFG, the resistance of poly(PBA-*ran*-PDMS)/VAFG sensor tends to be stable at 90 ℃ as the increase of temperature, which indicates that temperature has a restraining effect on the resistance. **Figure S8c** shows the resistance pressure curve of poly(PBA-*ran*-PDMS)/VAFG sensor under different pressures. And the resistance of poly(PBA-*ran*-PDMS)/VAFG changes significantly with the increase of pressure, indicating its excellent strain sensitivity. In order to verify the pressure change stability, cyclic resistance tests were performed on the resistance at different pressures, and the results showed that the resistance remained stable at different pressures and the poly(PBA-*ran*-PDMS)/VAFG sensor had excellent pressure stability.

Heat conduction characteristics of the poly(PBAx-*ran*-PDMS)/VAFG architecture were investigated by transient-state COMSOL multiphysics thermal simulation. The model simulates the heat dissipation of the model of VAFG, consisting of a heat source representing the bottom block component, poly(PBAx-*ran*-PDMS)/VAFG as the heat dissipating middle block, and a heat sink part at the top. According to the direction of heat flow, it was used to load a heat source with a constant temperature of 50 ˚C on the marked surface, the plane of the heat transfer end is set to 10 ℃, which with substeps of 20 steps totaling 0.1 s. The composite material and structural parameters of the modelled system are shown in **Table S1**.

Table S1. The composite material and structural parameters of the modelled system.

**Material**	**Density (kg m^{-3})**	**Specific heat capacity (J kg^{-1} ˚C^{-1})**	**Thermal conductivity (*k*, W m^{-1} K^{-1})**
Poly(PBA-*ran*-PDMS)	140	1000	0.2
VAFG	1500	700	2000
Air	0.00129	1003	0.023

Table S2. Summary of data on adhesion of materials reported in the literature.

**Materials**	**Adhesion strength (N/m)**	**Ref.**
PBA–UPy7.2	57	1
DCPD-DNE (10 wt%)	2800	2
polyurethane	2500	3
ASHA-C1	5000	4
PDMS	4000	5
PDMS	1800 ± 210	6
DST	600	7
LCE	3700	8
PVA/PA	2000	9
DAE-3	2000	10
MREs	4640	11
PTCM-Gly5	125	12

Table S3. Theoretical and experimental in-plane thermal conduction properties as function of compression ratio.

Num.	Material	K// (W/mK)	K⊥ (W/mK)	Filler Content	Tensile strength (MPa)	Ref.
1	GnP-polymer		12.4	48.5 wt%	0.3	13
2	rLGO/PVDF-HFP	19.5		27.2 wt%	0.8	14
3	epoxy/GnP		2.13	6 vol%	-	15
4	PP/PDA/G	2.73	10.93	27 vol%	0.192	16
5	GNs/GF/NR	11.16	0.15	10.27 vol%	0.7	17
6	PDA-rGO/SR		1.50	1.46 wt%	-	18
7	PVDF/GnP		13	50 wt%	0.192	19
8	graphene/TPU		12	45 wt %	11.3	20
9	PA6/POE-g-MAH/PS/graphite	3.24	5.5	50.0 wt %	2.6	21
10	PE/graphene	3.43	3.43	15 vol %	-	22
11	PLA/graphene	~1	~1	12 wt %	0.1565	23
12	MC/GN	0.79	0.79	1 wt %	83	24
13	Al/GNPs	224		1 wt %	-	25
14	EP/GR-Fe_3O_4	0.28		66.6 wt %	-	26
15	CNF/GP	5.46	0.07	10 wt %	30	27
16	PEG/MXene/GR		1.64	7.35 wt %	8	28
17	EP-$BaTiO_3$-$GSiC_{nw}$	0.64	0.64	0.092 vol%	20	29
18	PVA/GO	~2.5		5 wt %	56	30
19	PVDF/CNT/GNP	1.92	1.92	20 wt %	5.3	31
20	Poly(PBA-*ran*-PDMS)/VAFG	3.6	13.4	2.17 wt%	2.23	This work

Table S4. The coefficients of the fitting equation.

	T-s			***R/R*₀*-P***		
	A	***B***	***C***	***A***	***B***	***C***
Stainless steel	-0.0139	1.064	28.02	14.19	-57.28	91.49
Glass	-0.087	0.731	26.36	11.41	-46.28	93.11
Wood	-0.048	0.44	26.15	10.17	-42.61	96.11
Rubber	-0.002	0.247	25.52	7.73	-31.05	99.9

Reference

1 M. Shtein, R. Nadiv, M. Buzaglo, K. Kahil, O. Regev, *Chem. Mater.* **2015**, 27, 2100.
2 P. Kumar, S. Yu, F. Shahzad, S. M. Hong, Y.-H. Kim, C. M. Koo, *Carbon* **2016**, 101, 120.
3 Meng Q, Han S, Araby S, Zhao Y, Liu Z, Lu S. *J. Adhesion Sci. Technol.* **33**, 1337-1356 (2019).
4 N. Song, D. Cao, X. Luo, Q. Wang, P. Ding, L. Shi, *Compos. Part A: Appl. Sci. Manufacturing* **2020**, 135, 105912.
5 F. An, X. Li, P. Min, H. Li, Z. Dai, Z.-Z. Yu, *Carbon* **2018**, 126, 119.
6 S. Song, J. Wang, C. Liu, J. Wang, Y. Zhang, *Nanoscale* **2019**, 11, 15234.
7 M. Clausi, S. Grasselli, A. Malchiodi, I. S. Bayer, *Appl. Surface Sci.* **2020**, 529, 147070.
8 H. Guo, H. Zhao, H. Niu, Y. Ren, H. Fang, X. Fang, R. Lv, M. Maqbool, S. Bai, *ACS nano* **2021**, 15, 6917.
9 Y. Jia, H. He, Y. Geng, B. Huang, X. Peng, *Compos. Sci. Technol.* **2017**, 145, 55.
10 J. Jing, Y. Chen, S. Shi, L. Yang, P. Lambin, *Chem. Engineering J.* **2020**, 402, 126218.
11 R. Kotsilkova, E. Ivanov, V. Georgiev, R. Ivanova, D. Menseidov, T. Batakliev, V. Angelov, H. Xia, Y. Chen, D. Bychanok, *Polymers* **2020**, 12, 1208.
12 A. Faghihnejad, K. E. Feldman, J. Yu, M. V. Tirrell, J. N. Israelachvili, C. J. Hawker, E. J. Kramer, H. Zeng, *Adv. Funct. Mater.* **2014**, 24, 2322.
13 G. O. Wilson, M. M. Caruso, S. R. Schelkopf, N. R. Sottos, S. R. White, J. S. Moore, *ACS Appl. Mater. Interfaces* **2011**, 3, 3072.
14 A. Feula, X. Tang, I. Giannakopoulos, A. M. Chippindale, I. W. Hamley, F. Greco, C. P. Buckley, C. R. Siviour, W. Hayes, *Chem. Sci.* **2016**, 7, 4291.
15 Z. Zhang, N. Ghezawi, B. Li, S. Ge, S. Zhao, T. Saito, D. Hun, P. F. Cao, *Adv. Funct. Mater.* **2021**, 31, 2006298.
16 S. Jo, H. Lee, H. Jang, D. R. Kim, *ACS Appl. Mater. Interfaces* **2021**, 13, 13684.
17 S. Kaur, A. Narayanan, S. Dalvi, Q. Liu, A. Joy, A. Dhinojwala, *ACS Central Sci.* **2018**, 4, 1420.

18 H. Yuk, C. E. Varela, C. S. Nabzdyk, X. Mao, R. F. Padera, E. T. Roche, X. Zhao, *Nature* **2019**, 575, 169.
19 T. Ohzono, M. O. Saed, E. M. Terentjev, *Adv. Mater.* **2019**, 31, 1902642.
20 R. Hu, J. Zhao, Y. Wang, Z. Li, J. Zheng, *Chem. Engineering J.* **2019**, 360, 334.
21 Z. Xu, L. Chen, L. Lu, R. Du, W. Ma, Y. Cai, X. An, H. Wu, Q. Luo, Q. Xu, *Adv. Funct. Mater.* **2021**, 31, 2006432.
22 R. Li, D. Wang, P.-a. Yang, X. Tang, J. Liu, X. Li, *Industrial Engineering Chem. Research* **2020**, 59, 9143.
23 Y. Wei, L. Xiang, H. Ou, F. Li, Y. Zhang, Y. Qian, L. Hao, J. Diao, M. Zhang, P. Zhu, *Adv. Funct. Mater.* **2020**, 30, 2005135.
24 Y. Zhuang, X. Cao, J. Zhang, Y. Ma, X. Shang, J. Lu, S. Yang, K. Zheng, Y. Ma, *Compos. Part A: Appl. Sci. Manufacturing* **2019**, 120, 49.
25 A. Saboori, M. Pavese, C. Badini, P. Fino, *Acta Metallurgica Sinica (English Letters)* **2017**, 30, 675.
26 Z. Wu, J. Chen, Q. Li, D.-H. Xia, Y. Deng, Y. Zhang, Z. Qin, *Mater.* **2021**, 14, 2013.
27 D. Jiao, N. Song, P. Ding, L. Shi, *Compos. Commun.* **2022**, 101101.
28 L. Jin, P. Wang, W. Cao, N. Song, P. Ding, *ACS Appl.Mater. Interfaces* **2021**.
29 J. He, H. Wang, Z. Su, Y. Guo, X. Tian, Q. Qu, Y.-L. Lin, *Compos. Part A: Appl. Sci. Manufacturing* **2019**, 117, 287.
30 X. Pan, M. G. Debije, A. P. Schenning, C. W. Bastiaansen, *ACS Appl. Mater. Interfaces* **2021**, 13, 28864.
31 Y.-j. Xiao, W.-y. Wang, X.-j. Chen, T. Lin, Y.-t. Zhang, J.-h. Yang, Y. Wang, Z.-w. Zhou, *Compos. Part A: Appl. Sci. Manufacturing* **2016**, 90, 614.
